# Identification and characterization of small RNAs expressed by *Leptospira borgpetersenii* serovar Hardjo and their conservation in the genus *Leptospira*

**DOI:** 10.1186/s12866-026-05052-1

**Published:** 2026-04-18

**Authors:** Ellie J Putz, Luis GV Fernandes, Darrell O Bayles, Bienvenido W Tibbs-Cortes, Bruna Petry, Sathesh K Sivasankaran, Jarlath E Nally

**Affiliations:** 1 Infectious Bacterial Disease Research Unit, USDA Agriculture Research Service, National Animal Disease Center, Ames, IA USA; 2 Food Safety and Enteric Pathogens Research Unit, USDA Agriculture Research Service, National Animal Disease Center, Ames, IA USA; 3 Genome Informatics Facility, Iowa State University, Ames, IA USA

**Keywords:** *Leptospira borgpetersenii*, RNAseq, Small RNAs

## Abstract

**Background:**

Leptospirosis is a global zoonotic disease affecting humans, companion animals, and domestic livestock. Clinical presentation of disease is dependent on complex interactions between host species and pathogenic species, serovars, and strains of *Leptospira*. This is exemplified by *L. borgpetersenii* serovar Hardjo strains HB203 and JB197, which differ very little by genome content but cause divergent clinical disease phenotypes in the hamster model of leptospirosis. JB197 causes an acute lethal disease while HB203 establishes a chronic asymptomatic infection of the kidneys. Previous work has shown that *Leptospira* modify their gene expression in response to temperature.

**Results:**

RNA sequencing performed on serovar Hardjo strains HB203 and JB197 cultured at both 29˚C and 37˚C directly from experimentally infected hamsters identified 266 small RNAs (sRNAs) previously unannotated for *L. borgpetersenii*. Levels of expression for each novel candidate sRNA are reported: 67 and 10 sRNAs were differentially expressed between strains (at 29˚C and 37˚C respectively) while 32 and 10 were differentially expressed between temperature conditions (within JB197 and HB203 respectively). Conservation analysis of candidate sRNAs within the genus *Leptospira* identified a binomial distribution comprising highly conserved sRNAs such as JB_C1_N135/*LIC1nc80* and JB_C1_N610 which are found in 42/43 pathogenic species compared to others such as JB_C1_N595 and JB_C1_N1040 which are restricted to strains of *L*. *borgpetersenii*.

**Conclusions:**

The nuances of what contributes to *Leptospira* strain-specific pathogenesis are poorly understood. Non-coding sRNAs have regulatory impacts on gene expression that can alter leptospire protein profiles, virulence factors, and host-pathogen interactions. Levels of conservation of these sRNAs within the genus *Leptospira* identified those that were highly conserved compared to others that were species-specific. Results emphasize the broad role that sRNAs play in regulating gene expression of *Leptospira*, and the potential for sRNAs to be used for clinical detection as well as species/serovar specific typing.

**Supplementary Information:**

The online version contains supplementary material available at 10.1186/s12866-026-05052-1.

## Background

Leptospirosis is a zoonotic infection caused by pathogenic bacteria of the genus *Leptospira* which infects humans, companion animals, and livestock around the world. Host species often interact variably with different species, serovars, and strains of *Leptospira* and develop a wide range of clinical signs and disease severity [1]. In cattle, leptospirosis causes abortion and reproductive losses, with *L. borgpetersenii* being the most prominent species colonizing bovine hosts [2]. In the central United States, 7.2% of abattoir cattle were found to be shedding *L. borgpetersenii* via urine [3]. In other parts of the world, *L. borgpetersenii* can be a significant cause of human disease [4]. Transmission can occur directly from infected urine or indirectly through contaminated water or soil. As such, *Leptospira* may encounter substantial variation in temperature during transmission, ranging from host core temperatures (closer to 37˚C) to those of warm environments (closer to 29˚C). Notoriously fastidious to culture in the laboratory, *Leptospira* have historically only been successfully cultured at 29˚C, but a new medium has recently facilitated their culture at 29˚C and 37˚C directly from mammalian hosts [5], including humans [6], mice [7], rats [8], mongoose [9], red panda [10], dogs (unpublished data), cattle [11–13], and horses [14], as well as new species from environmental sources including soil [15] and water [16].

While commercial bacterin vaccines are available to livestock producers, they suffer from poor cross protection among serovars and a lack of cross protection amongst serogroups [17]. As a result, bacterin vaccines contain numerous serovars, often tailored to the host species and region where the vaccine will be deployed. While the leptospirosis research field emphasizes serovar characterization, evidence has shown substantial variation in gene and protein expression at the strain level of leptospiral distinction [18–22]. This is well modeled by *L. borgpetersenii* serovar Hardjo strains JB197 and HB203, which although nearly identical by gene content, cause severe acute versus asymptomatic chronic disease, respectively, in the hamster model of infection [23]. In-depth transcriptomic and proteomic investigations into profile differences of JB197 and HB203 have been conducted, characterizing their differences when cultured at 29˚C and 37˚C [19–22]. Notably, these efforts have established that both strain and temperature can vastly alter the expression of outer membrane surface proteins and virulence factors that primarily interact with the host immune system. For instance, the expression of leptospiral immunoglobulin-like (Lig) protein B (LigB) is significantly higher in strain HB203 compared to JB197 [19]. Further, *ligB* has increased expression at 29˚C versus 37˚C in HB203, but the inverse is true for JB197 which has higher *ligB* expression at 37˚C versus 29˚C. LigB has been established as an immunogen and a target for vaccine design and mutagenesis [24, 25], but its gene and protein expression are heavily influenced by strain and temperature [19, 22]. Other outer membrane proteins including LipL41, LipL32, LipL45, and LipL21 display similar strain and condition sensitivities [19, 22]. This indicates that a bacterin vaccine could elicit varying degrees of protection depending on the strain and growth conditions used to produce it.

To understand strain and temperature sensitivity, it is critical to investigate the potential mechanisms responsible for regulating protein expression. One mechanism is post-transcriptional regulation by non-coding small RNAs (sRNAs). sRNAs are involved in a myriad of bacterial processes with regulatory roles affecting physiology, metabolism, and virulence [26–28]. Often, sRNAs act as activating or inhibiting agents for their respective mRNA targets by influencing binding, blocking, and/or conformational changes depending on base pair binding or utilization of a chaperone [28, 29]. sRNAs can be classified as either *cis* or *trans*-encoded based on their genomic proximity to the loci of their targets. Among the *cis-*acting sRNAs, antisense sRNAs are encoded in the non-coding strand of a gene, and the resultant complementarity enables interaction with the mRNA of the transcribed gene. Many other sRNAs, however, are not encoded proximal to the locus of their target and act in *trans*. These *trans*-acting sRNAs have been found to originate as distinct intergenic transcripts, but research has also identified *trans*-acting sRNAs derived from 3’ and 5’ UTRs [30–32]. Although less characterized than other Gram-negative bacteria, within *Leptospira*,* L. interrogans* sRNAs have been shown to be differentially expressed between culture and dialysis membrane chamber conditions in the rat model [33] as well as between temperatures [34] and in response to oxidative stress [35].

In this work, we analyzed high throughput sequencing data from strains JB197 and HB203 cultured at different temperatures and identified 266 previously unannotated candidate sRNAs for *L. borgpetersenii*. Many of these sRNAs were differentially expressed within strain and/or in response to temperature. Given that the genus *Leptospira* comprises 74 species which are subdivided into pathogens (43 species) and saprophytes (31 species) [36], we then determined the level of conservation of these sRNAs. Characterizing the mechanisms of gene regulation in *Leptospira* is critical to understanding the pathogenic mechanisms of infection.

## Methods

### Experimental samples, RNA isolation and sequencing

The RNAseq dataset was generated in a previous study using four biological replicates of *L. borgpetersenii* serovar Hardjo strain HB203 and four biological replicates of *L. borgpetersenii* serovar Hardjo strain JB197 isolated from infected hamsters [19] directly into HAN media at 29 and 37˚C [5].

### Manual curation of sRNAs using Integrated Genome Browser (IGB)

IGB was used to visually inspect RNAseq data and identify areas of expression that did not map to the annotated genome features. Candidate unannotated sRNAs had to meet requirements of at least 250 reads in a minimum of two replicates under at least one condition. sRNAs were classified as having either sense, antisense, intergenic, 3’ UTR, or 5’ UTR configurations. Both HB203 and JB197 reads were mapped to the *L. borgpetersenii* JB197 reference genome (RefSeq ID: chromosome 1: NC_008510.1, chromosome 2: NC_008511.1). To account for HB203-specific sRNAs that may not be expressed in JB197, HB203 reads were also evaluated by mapping to the HB203 reference genome (RefSeq ID: chromosome 1: NZ_CP021412.1, chromosome 2: NZ_CP021413.1). Except for sRNAs which were only found in HB203, the coordinates for each sRNA are the coordinates of that sRNA in the JB197 genome.

### Differential expression of sRNAs

Previous work examined the differential expression (DE) of annotated genes across strains JB197 and HB203 cultured at 29 and 37˚C [19]. This DE analysis was repeated using DESeq2 to incorporate the 266 novel sRNAs identified and annotated in this work. Analysis was conducted as previously described [19, 37] with an emphasis on identifying DE among newly annotated sRNAs; Differential expression (DE) analysis was completed in R (v3.6.1; https://www.r-project.org/) using the DESeq2 package (v1.29.0 [37]). Briefly, linear regression models were fit in DESeq2 independently for each condition of interest (JB197 37˚C vs. 29˚C, HB203 37˚C vs. 29˚C, 29˚C JB197 vs. HB203, and 37˚C JB197 vs. HB203). Significance was determined if normalized counts exceeding ten for at least one condition, the adjusted p-value < 0.05, and there was a minimum fold change (FC) of three (Log2FC ≥ 1.585).

### Homology and conservation

All complete and draft Leptospiral whole genome assemblies available from NCBI (1054 genomes on June 2, 2025) were downloaded and formatted into a Leptospiral-genome comprehensive BLAST database. Homology determinations were made by using GLASSgo [38] to query each sRNA against the comprehensive BLAST database. A local instance of GLASSgo was run in an Apptainer container using the GLASSgo defaults and the 52% homology cutoff. GLASSgo output the identified homologs along with the percent identity (PI) of the homolog cluster found to contain the sRNA. The minimum, average, and median PIs for each sRNA were calculated over all Leptospires and at each NCBI taxonomic ID level. The NCBI common taxonomic tree was downloaded from NCBI and the presence of homologs was highlighted on each taxonomic ID branch if GLASSgo reported a homolog for that branch. Tree-based visualizations were generated with R (R Core Team, 2025) and the following R-packages: ape, ggplot2, ggtree, treeio, tidytree, and tidyverse [39–44].

### RNA isolation for Northern blots

Triplicate cultures of *L. borgpetersenii* serovar Hardjo strain TC129 [21], serovar Arborea strain LR131 [7, 45], and *L. interrogans* serovar Canicola strain LAD-1 [46, 47] and serovar Copenhageni strain R47 [8] were propagated in HAN media at 37 °C with 5% CO_2_. The cells were grown until the mid-late log phase of growth (~ 1–3 × 10^8^ cells per mL) and were then harvested by centrifugation at 10,000 ×*g* for 30 min at 4 °C. Pellets were immediately stored at -80 °C until extraction of RNA using the TRIzol method as previously described [48]. Briefly, 1 mL of TRIzol (Invitrogen, CA, USA) was added to frozen pellets and gently pipetted until thawed. The samples were vortexed and incubated at room temperature for 10 min before adding 260 µL of chloroform. After vigorous shaking, the samples were incubated for another 10 min at room temperature. Phase separation was achieved by centrifugation at 12,000 × *g* for 10 min at 4 °C, and the aqueous phase was collected. To precipitate nucleic acids, 660 µL of isopropanol was added, followed by a 10-minute incubation at room temperature. The samples were then centrifuged at 12,000 × *g* for 10 min at 4 °C, and the resulting pellets were washed with 75% ethanol. After drying for 30 min in a SpeedVac, the pellets were resuspended in 50 µL of RNase-free water and incubated at 55 °C for 10 min. Samples were quantified via the Qubit HS RNA assay, and their integrity was verified by 1% agarose gel electrophoresis.

### Northern blots

Five hundred nanograms of RNA from each sample were mixed with one volume of 2X denaturing loading buffer containing 95% formamide (NEB) and incubated for 5 minutes at 95°C. Low range ssRNA ladder (NEB) was diluted to a final volume of 0.1 µL per well and denatured in 2X RNA loading buffer as above. After denaturation, samples and ladder were incubated on ice for 2 minutes before being loaded onto a 10% polyacrylamide tris borate EDTA (TBE)-urea gel and fractionated at a constant voltage of 150 V in 1X TBE. The gel was subsequently stained in 1.5X SYBR Safe and visualized briefly to confirm RNA fractionation and integrity. Next, RNA was transferred to a BrightStar-Plus positively charged nylon membrane (Ambion) in 0.5X TBE for 1.5 hours at a constant voltage of 10 V at 4°C. The membrane was allowed to air dry, and RNA was UV cross-linked to the membrane by a Stratalinker UV 1800 Crosslinker (Stratagene) using the “autocrosslink” mode (120,000 microjoules, 254 nm). Bands were then visualized by UV light. Sections of membrane were then placed in 50-mL conical tubes containing 9 mL ULTRAhyb-Oligo hybridization buffer (Ambion) and incubated for 30 minutes at 45 or 60°C in a rotation chamber. 1 mL of ULTRAhyb-Oligo buffer containing 1 µL of 100 µM dual biotinylated RNA probe (IDT) was heated at 95°C for 5 minutes and added to the conical tube for a final concentration of 10 nM probe. The following probes were used: a positive control probe targeting the highly expressed LBJ_RS18060 RNase P gene *rnpB* (5’ TTACCCCGCCATTTCACCCTTACC 3’), a negative control probe antisense to *rnpB* (5’ GGTAAGGGTGAAATGGCGGGGTAA 3’), and a probe targeting JB_C1_N135/ *LIC1nc80* (5’ GACT**C**TTCCTCC**G**CGTTACTAACG 3’). Membranes were then incubated with the probe in a rotation chamber overnight. The next day, the membranes were subjected to two 5-minute washes with 5 mL 2X SSC buffer with 0.1% SDS followed by two 15-minute washes with 5 mL 0.1X SSC buffer with 0.1% SDS. Hybridized probes were visualized by incubation with horseradish peroxidase-conjugated streptavidin and chemiluminescent substrate using the Chemiluminescent Nucleic Acid Detection Module (Thermo Scientific). Blots were visualized using a Chemidoc MP and associated ImageLab software (BioRad) using the “Chemi Hi Resolution” application.

## Results

### Identification of candidate sRNAs in *L. borgpetersenii* serovar Hardjo strains JB197 and HB203

After evaluation of transcriptomic data from *L*. *borgpetersenii* serovar Hardjo strains JB197 and HB203 using the Integrated Genome Browser (IGB), 266 candidate sRNAs were identified by manual curation. As their genomes are over 99% identical by nucleotide sequence, reads from both conditions, for HB203 and JB197, were mapped to the JB197 reference genome, yielding 216 previously unannotated sRNAs in at least one of the experimental conditions: JB197 29˚C, JB197 37˚C, HB203 29˚C, HB203 37˚C. To ensure all sRNAs from HB203 were also identified, HB203 reads were mapped to the HB203 reference genome and manually curated where an additional 50 unannotated sRNAs were identified. A breakdown of sRNA by strain and type is available in Table 1. For all identified sRNAs, the genomic coordinates and sequences, length, conditions under which expression occurred, classifications, and the presence and location of TATA boxes appear in Supplementary Table 1. Of the 266 sRNAs identified, 97 were less than 200nt, 144 were 200 to 500nt and 25 were greater than 500nt. In addition to their coordinates, sRNAs were labelled as sense, antisense, intergenic, and/or in 3 or 5’ UTR configurations with co-located annotated genes (Table 1, Supplementary Table 1).

**Table 1:** Summary of sRNA description: Candidate sRNAs were labelled as sense, antisense, intergenic, and/or in 3 or 5’ UTR configurations with co-located annotated genes. Of the 266 total sRNAs, 50 were uniquely annotated using the HB203 genome, while all others were mapped to the JB197 genome. sRNAs may have more than one label as listed in Supplementary Table 1

sRNA label	Annotated in HB203	Annotated in JB197
Sense	16	79
Antisense	35	137
Intergenic	11	58
3’	8	53
5’	9	41

**Fig. 1:**
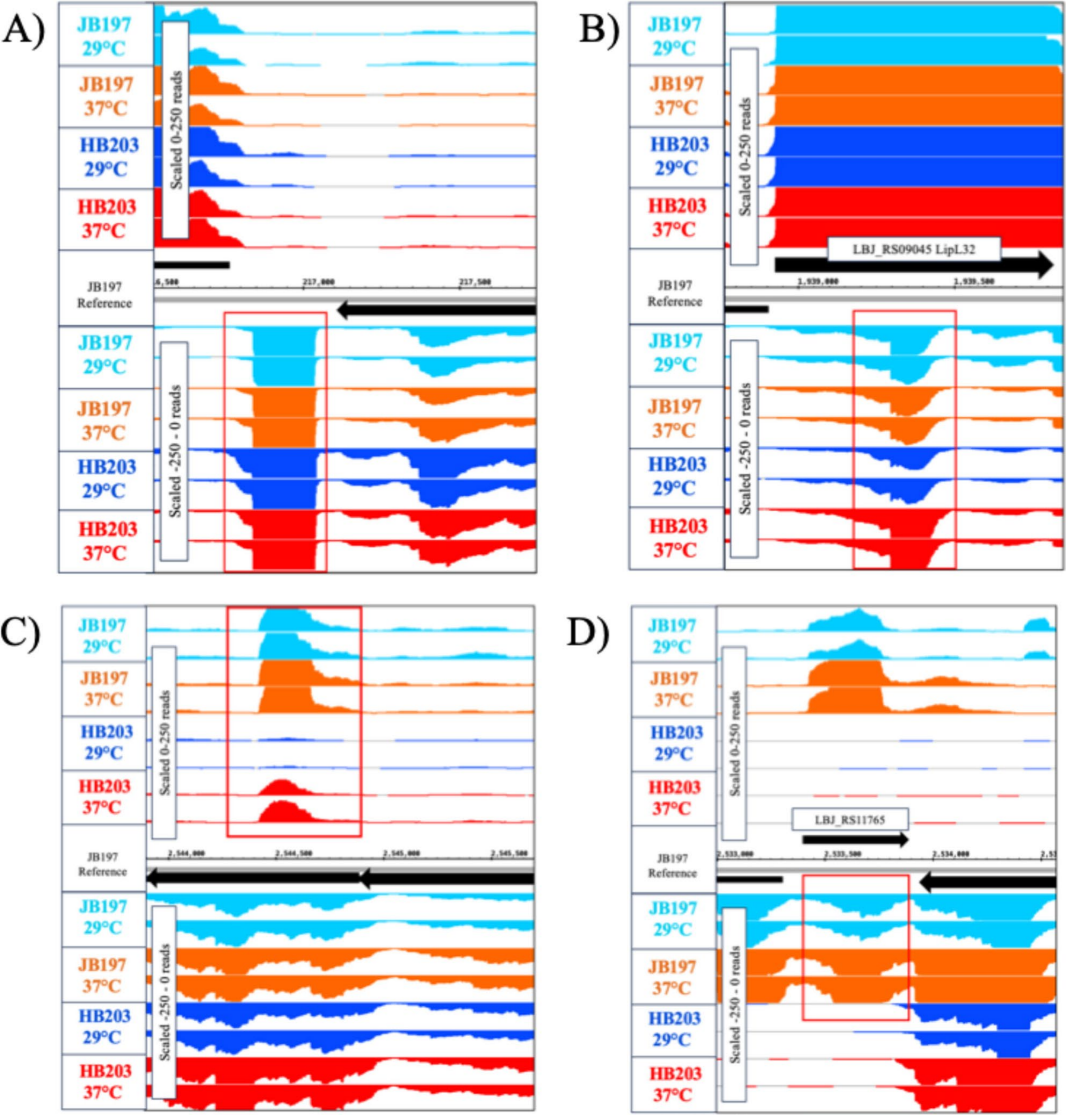
Integrated Genome Browser (IGB) manual curation of candidate sRNAs. IGB detected sRNAs (surrounded by red square) with normalized read counts of given conditions: JB197 29˚C (light blue), JB197 37˚C (orange), HB203 29˚C (dark blue), and HB203 29˚C (red). Designated sRNA had to meet a criteria of a minimum of 250 reads in at least one condition and were organized into sense, antisense, intergenic, 3’ or 5’ UTR configurations. Two of four biological replicates are shown. Strain specific sRNAs were detected and differential expression was identified amongst conditions of interest. **A** JB_C1_N135/*LIC1nc80*; (**B**) JB_C1_N610, anti-sense to LipL32; (**C**) JB_C1_N795, an antisense sRNA with differential expression between strains and within strains between temperatures; (**D**) JB_C1_N790 which shows gene and sRNA expression only in JB197

**Fig. 2:**
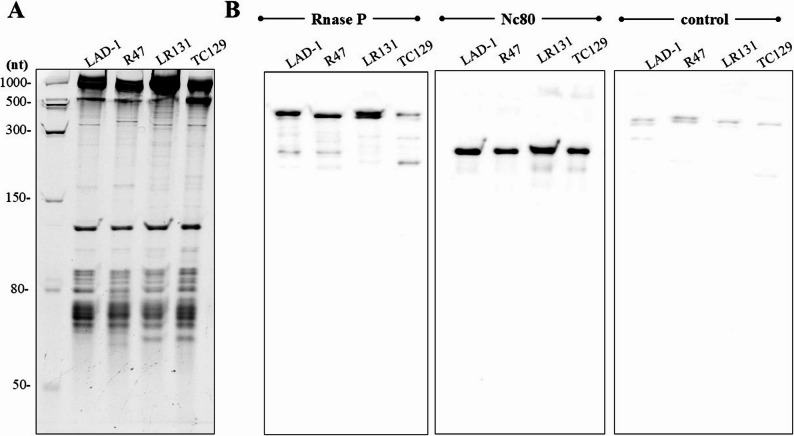
Northern blot of JB_C1_N135/*LIC1nc80* highlights conservation of sRNAs identified by manual analysis of RNAseq read mapping data. **A** Representative polyacrylamide gel showing the total RNA samples from *L. interrogans* serovar Canicola strain LAD-1, serovar Copenhageni strain R47, and *L. borgpetersenii* serovar Arborea strain LR131, and serovar Hardjo strain TC129. **B** Blot showing the transcription of RNase P and JB_C1_N135/*LIC1nc80* (hybridization at 45 °C), both at the predicted sizes. An antisense RNase P probe was used as a negative control (hybridization at 45 °C). Reactive bands were visualized by incubation with chemiluminescent substrate and membranes were exposure for 30 s

Depictions of representative curated sRNAs within IGB can be seen in Fig. 1. An sRNA orthologous to JB_C1_N135 (Fig. 1A) has been previously identified as *LIC1nc80* in *L*. *interrogans* [33] where it exhibits differential expression in response to diverse environmental and host-associated cues. Similarly, JB_C1_N135/*LIC1nc80* was differentially expressed among the conditions of interest in the present study, being more highly expressed in HB203 than JB197 at 29˚C (log2(foldchange) of -1.92, adjusted p-value = 2.65 × 10^− 19^) (Table 2, Supplementary Table 2). The conserved expression of sRNA JB_C1_N135/*LIC1nc80* in two different species and four different serovars of *Leptospira* was further apparent by Northern blot (see Fig. 2). For JB_C1_N135/*LIC1nc80* detection, probe hybridization at 60 °C resulted in transcript detection exclusively in *L. borgpetersenii* strains (Supplementary Fig. 1). When the hybridization temperature was lowered to 45 °C, transcripts were also detected in *L. interrogans* strains (Fig. 2B). This difference is likely due to the probe being designed based on the *L. borgpetersenii* sequence, which contains two nucleotide mismatches (highlighted as bold nucleotides in the probe sequence above) when compared to the *L. interrogans* sequence, leading to *LIC1nc80* detection only under less stringent conditions in the latter species.

While sRNAs have the potential to influence the expression of target mRNAs in a myriad of ways, binding/blocking and conformational changes are common to sRNAs interacting with nearby genes. Therefore, special attention was paid to previously unannotated sRNAs around known immunogenic genes and/or virulence factors. Figure 1B denotes a DE sRNA (JB_C1_N610) antisense to LipL32, the robustly expressed membrane protein used for most diagnostics to identify pathogenic leptospires (which itself was differentially expressed in this data set). The experimental design examining strain and temperature differences between JB197 and HB203 cultured at 29˚C and 37˚C also places an emphasis on sRNAs showing conditional expression. Figure 1C depicts JB_C1_N795, an antisense sRNA with differential expression between strains and within strains between temperatures. Further, some sRNAs identified only existed in certain strains or temperatures such as JB_C1_N790 (Fig. 1D) which shows gene and sRNA expression only in JB197 and not in HB203.

### Differential expression of sRNAs in strains JB197 and HB203 cultured at 29˚C and 37˚C

Our group has previously demonstrated robust transcriptomic variation by *L*. *borgpetersenii* strains JB197 and HB203 in response to temperature [19], an environmental cue also used by *L*. *interrogans* to regulate gene and protein expression in response to temperature changes encountered during host infection [49–51]. To analyze sRNA differential expression, previously collected transcriptomic data were analyzed for each of the four comparisons of interest (JB197 37˚C vs. 29˚C, HB203 37˚C vs. 29˚C, 29˚C JB197 vs. HB203, and 37˚C JB197 vs. HB203) including the newly annotated sRNA candidates (see Supplementary Table 2).

For 29˚C JB197 vs. HB203, 67 sRNAs were significantly DE (Log2FC > ± 1.585; adjusted p-value < 0.05); 58 up-regulated and 9 down-regulated. For 37˚C JB197 vs. HB203, a total of 10 sRNAs were DE with 4 positive fold changes, and 6 with negative fold changes (more greatly expressed in HB203 vs. JB197). Within strain JB197 37˚C vs. 29˚C, there were 32 significant DE sRNAs; 4 with positive fold changes, and 28 with negative fold changes (greater expression at 29˚C compared to 37˚C). For HB203 37˚C vs. 29˚C, there were 10 DE sRNAs; 8 with positive fold changes and 2 with negative fold changes (greater expression at 29˚C compared to 37˚C). The details of these sRNAs are detailed in Supplementary Table 2.

**Fig. 3:**
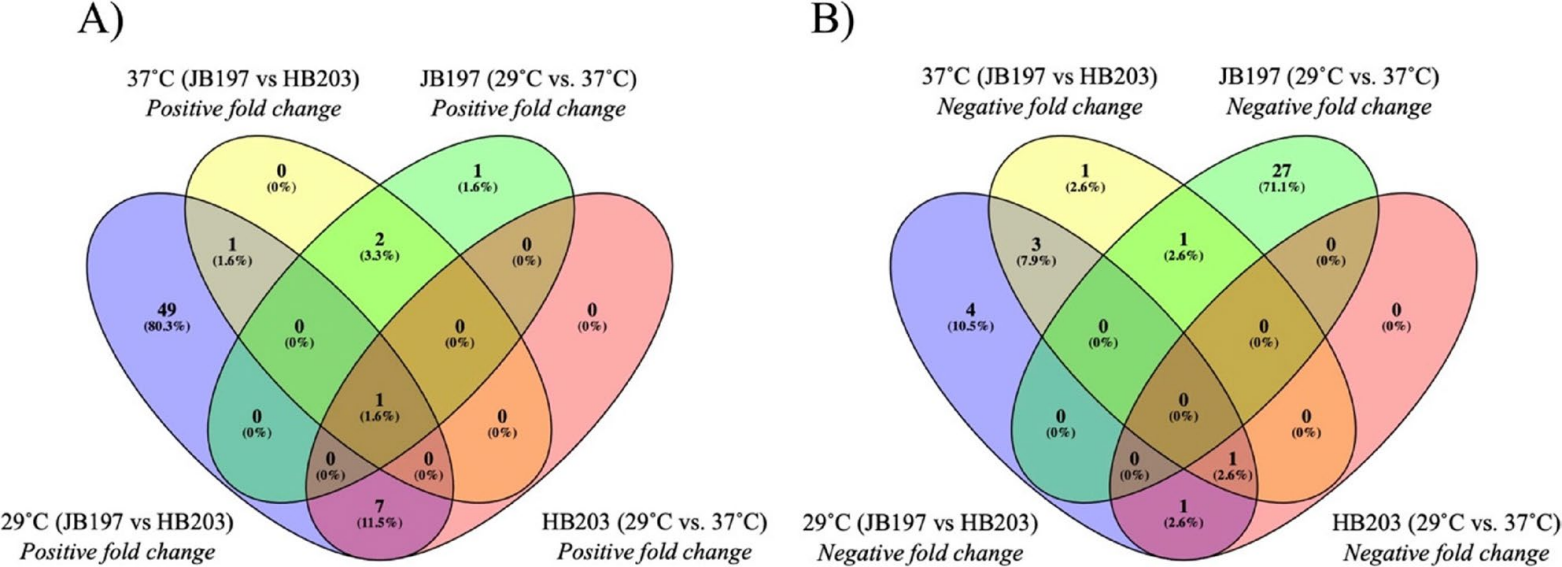
Venn diagrams detailing differentially expressed sRNA in conditions of interest. Depicted are the number of significantly DE sRNAs in JB197 (29˚C vs. 37˚C, green), HB203 (29˚C vs. 37˚C, red), 29˚C (JB197 vs. HB203, blue), and 37˚C (JB197 vs. HB203, yellow). Panel (**A**) denotes significantly DE sRNAs with positive fold changes, and panel (**B**) denotes the sRNAs with negative fold changes

To determine distribution and evaluate whether sRNAs amongst experimental conditions showed conserved DE, a Venn diagram was constructed looking at positive and negative fold change significantly DE sRNAs (Fig. 3). While the sRNAs did not largely exhibit conserved DE, the sRNA JB_C1_N595 was significantly DE with a positive fold change across all conditions. JB_C1_N595 is an antisense sRNA (antisense to LBJ_RS08925, *flhB* coding an inner membrane protein) expressed more in JB197 and more at 37˚C in both strains.

### Conservation of leptospiral sRNAs

**Fig. 4:**
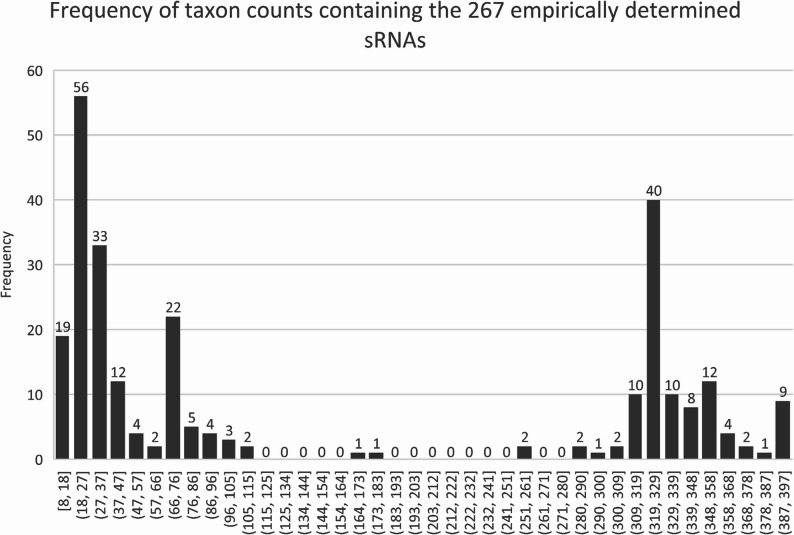
Binomial Distribution of conservation of novel sRNAs. The x-axis represents a windowed count of the 397 NCBI taxonomic identifications that were queried for homologs of the 266 candidate sRNAs from this study. The y-axis represents the frequency (I.e. count) of how many of the sRNAs were identified within a number of taxonomic IDs

The identification of sRNAs that are expressed by strains of *L*. *borgpetersenii* serovar Hardjo, many of which are differentially expressed in response to environmental cues such as temperature, may reflect their role in regulating gene expression. Phylogenetically conserved sRNAs are more likely to be functionally significant and *L*. *borgpetersenii* is one of 43 pathogenic species in the genus *Leptospira*. Therefore the 266 candidate sRNA sequences were used to query a comprehensive database comprising 1054 unique *Leptospira* genome accessions, representing 397 unique NCBI leptospiral taxonomic identifications, using GLobal Automated sRNA Search go (GLASSgo) [38] to determine their presence and degree of conservation in other species/serovars/strains. Table 2 details ten representative sRNAs from our dataset that encompass a broad range of features of interest including sRNA type (intergenic, antisense, 5’ UTR), potential virulence involvement (upregulated in vivo, antisense to virulence factor genes), unique to strain (predominantly expressing in JB197 or HB203), and/or highly differentially expressed between strains and temperatures. This includes JB_C1_N135 (Fig. 1A) (previously identified as *LIC1nc80* in *L*. *interrogans* [33]) which was conserved in 948 genome accession hits with an average and median percent identify (PI) of 88.47 and 87.91, respectively (Table 2), and was identified in 42 of the 43 pathogenic species in the genus *Leptospira*, the exception being *L*. *gorisiae* (Supplementary Fig. 2). JB_C1_610 is antisense to the pathogenic specific gene *lipL32* and was also highly conserved; it was identified in 947 genome accession hits with an average and median PI of 91.26 and 98.02, respectively. In contrast, JB_C1_N595, which was more highly expressed by both JB197 and HB203 at 37 °C compared to 29 °C, was found in 185 genome accession hits, all of which were *L*. *borgpetersenii* except for 1 outlier found in *L*. *interrogans* which accounts for the single minimum PI value of 56.64 compared to the average and median PI of 93 and 90.71, respectively (Table 1 and Supplementary Fig. 1). Within *L*. *borgpetersenii*, JB_C1_N595 was identified in more than 10 serovars. We identified strain specific sRNAs including HB_C1_N1300 and HB_C1_N1320 in strain HB203 since these were not expressed in strain JB197. However, these sRNA sequences were detected in the genome of strain JB197 by GLASSgo (Supplementary Fig. 1). The frequency of taxon counts containing the 266 empirically determined sRNAs suggest three groupings: (1) those that are highly conserved in more than 300 taxa (*N* = 98), (2) those are limited to ≤ 47 taxa (*N* = 120) and (3) the remainder (*N* = 48) (Fig. 4). The taxonomic placements of sRNAs listed in Table 2 are shown in Supplementary Fig. 1. The complete dataset for degree of conservation for each sRNA listed in Table 2 is provided in Supplementary Table 3. The complete dataset for the taxonomic location of all candidate sRNAs is provided in the online repository Figshare at 10.6084/m9.figshare.30066829.

**Table 2:**
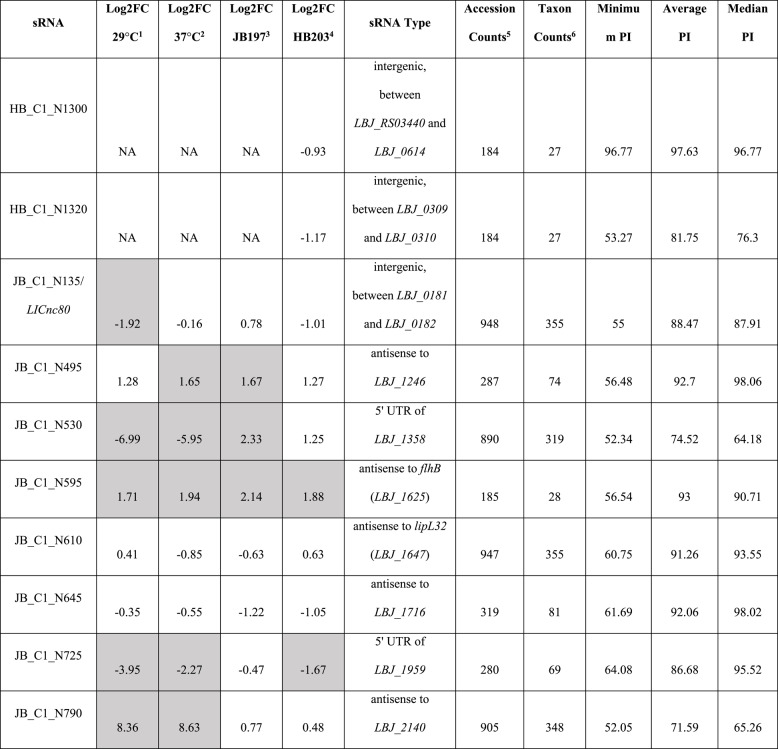
Characterization of representative candidate sRNAs. For a given comparison, the Log2 Fold Change (FC) of representative sRNAs of interest are reported. Significant comparisons (adjusted p-value < 0.05 and minimum absolute fold change ≥ 3/Log2FC ≥ 1.585) are highlighted in grey. For Log2(FC) of the sRNA in JB197 relative to HB203 at ^1^29 or ^2^37 °C positive fold change indicates higher expression in JB197. For Log2(FC) of the sRNA at 29 °C relative to 37 °C in ^3^JB197 or ^4^HB203 positive fold change indicates higher expression at 37 °C. For HB_C1_N1300 and HB_C1_N1320, these sRNAs were uniquely annotated in HB203 and thus differential expression analysis was conducted with counts mapped to the HB203 genome (all others mapped to JB197 genome). The number of homologous sRNAs detected in unique ^5^accessions and unique ^6^NCBI taxa. PI = Percent Identity

## Discussion

This work presents 266 previously unannotated candidate sRNAs for *L. borgpetersenii* serovar Hardjo. Our approach to identify sRNAs was based on the detection of expressed transcript that did not map to annotated genome features; this does not exclude non-regulatory types of small RNAs, such as riboswitch RNAs, CRISPR RNAs, or RNA processing RNAs. In addition, our method to isolate RNA is biased against RNAs smaller than 200 nucleotides though more than 35% of sRNAs identified were < 200 nucleotides in length (Supplementary Table 1). Further work revealed the strain and temperature specific differential expression of these sRNAs for strains JB197 and HB203 cultured at 29 °C and 37 °C. Significantly DE sRNAs were largely not shared across the comparisons of interest (JB197 37˚C vs. 29˚C, HB203 37˚C vs. 29˚C, 29˚C JB197 vs. HB203, and 37˚C JB197 vs. HB203). This collectively suggests that transcriptomic data is highly specific to strain and environmental temperature. The number of sRNAs manually identified in this work (*n* = 266) in *L. borgpetersenii* is similar to that identified by Zhukova et al. [34] using transcription start site mapping of *L. interrogans* serovar Manilae (*n* = 277 sRNAs at 30 °C and *n* = 226 at 37 °C) and by Tan et al. [52] using an in silico comparative genomics approach to study *L. interrogans* serovar Lai (*n* = 126 sRNAs at 30 °C).

The genus *Leptospira* is diverse and comprises 43 pathogenic species that can encounter a range of environmental cues during disease transmission and host infection. Leptospires modulate gene, protein and protein post-translational modifications during host infection [53–59], but much remains to be discovered about gene regulatory mechanisms. A phylogenetically conserved sRNA is more likely to be functionally significant and less likely a result of sequencing artifact or the result of noisy transcription. GLASSgo, which combines an iterative BLAST strategy with pairwise filtering and a graph-based clustering method utilizing RNA secondary structures, was used to identify conserved sRNAs in the genus. Of 266 candidate sRNAs identified, 98 were found in ≥ 300 taxon counts compared to 120 which were found in ≤ 47 taxon counts. Highly conserved sRNAs throughout the pathogenic species include JB_C1_N135/*LIC1nc80* and JB_C1_N610 (Table 1, Supplementary Table 3) and represent targets to investigate conserved gene regulatory mechanisms used by pathogenic species of *Leptospira*. In contrast, other sRNAs, such as JB_C1_N1040, are limited to a few serovars within the single species *L*. *borgpetersenii* (Supplementary Table 3) and may represent niche host-pathogen interactions. Alternatively, they may act as a unique signature potentially useful for classification purposes.

Future research needs include a deeper characterization of individual sRNA involvement in virulence. Recent work has demonstrated that RNAseP was differentially expressed in vivo versus in vitro in *L. interrogans* [33] and that mutations targeting RNAseP in the saprophyte *L*. *biflexa* are lethal [46]. Extension of such studies to examine the role of sRNAs in pathogenic species is now achievable with the recent development of mutagenesis capabilities for numerous leptospiral species [24, 47, 60–62].

Substantial work has established differential gene and protein expression in response to temperature by the model strains JB197 and HB203 of *L*. *borgpetersenii* [19, 21, 22]. This work, in addition to characterization of novel candidate sRNAs in *L. borgpetersenii*, adds insight into possible mechanisms of modulating gene and protein expression during infection. It is possible that sRNAs associated with virulence factors contribute to strain, serovar, and species variation which in turn may influence host versus pathogen interactions and vaccine efficacy. Further, sRNA-specific signatures may offer novel diagnostic targets of clinical relevance. As diagnostic methods improve and new species continue to be identified, the mapping, annotating, and characterizing of existing and new sRNAs will increase in importance.

## Conclusions

Leptospires, the atypical bacteria responsible for leptospirosis, are distributed world-wide in humans, companion and domestic animals, as well as the environment. In incidental hosts, severe disease can result in organ failure, and death while in asymptomatic reservoir hosts, bacteria typically colonize the kidneys and are excreted in the urine. Abortion is one clinical outcome in infected livestock. Different species, serovars, and even strains of leptospires may cause different presentations of disease in different host species which presents serious hurdles for the development of effective vaccines, diagnostics, and treatments. To characterize pathogen nuances, recent work has focused on genomic, transcriptomic, and proteomic characterization of leptospires. In this work, RNA sequencing identified for the first time the expression of small RNAs in two strains of *Leptospira borgpetersenii* serovar Hardjo. Many of these sRNAs are differentially expressed between the strains and in response to temperature. Within the novel candidate sRNAs characterized here, some were conserved across the genus *Leptospira*, while others were specific to the species and/or strain level. This highlights the potential for using sRNAs in the diagnostic detection of leptospirosis as well as for species and/or serovar specific discrimination. The identification and characterization of sRNAs is essential for understanding the pathogenic mechanisms of leptospirosis and how leptospires regulate gene expression during host infection.

## Supplementary Information

Supplementary Material 1: Supplementary Fig 1: Original images of Northern Blots presented in Figure 2.

Supplementary Material 2: Supplementary Fig. 2: NCBI taxonomic trees highlighting the presence of homologs for each sRNA listed in Table 2. The complete set of NCBI taxonomic trees highlighting the presence of homologs for all candidate sRNAs can be found at https://doi.org/10.6084/m9.figshare.30066829.

Supplementary Material 3: Supplementary Table 1: Detailed description of novel sRNAs. Table includes newly annotated name, whether the sRNA appeared on the minus (MI) or plus (PL) strand, start and ending coordinates, length of the sRNA, whether a TATA box was present (and if so its coordinates), the experimental conditions the sRNA was expressed in (JB197 29˚C, JB197 37˚C, HB203 29˚C, HB203 37˚C), whether it was possible the sRNA could be a UTR, its description (antisense (as), intergenic (igr), or on the 3’ or 5’ end), and its sequence. Coordinates of sRNAs found first in the HB203 transcriptomes (names beginning with “HB_C”) are specific to the HB203 genome so for column H (labelled expressed conditions), both refers to expression by HB203 at both 29 °C and 37 °C. All other sRNA genomic coordinates are given relative to the JB197 genome. Candidate sRNAs had to meet requirements of at least 250 reads in a minimum of two replicates under at least one condition.

Supplementary Material 4: Supplementary Table 2: Differential Expression of sRNAs among conditions of interest. Normalized read counts for all four biological replicates in two conditions (sheet labelled All) and differential expression contrasts for comparisons of JB197 29˚C vs. HB203 29˚C (sheet labelled 29), JB197 37˚C vs. HB203 37˚C (sheet labelled 37) and JB197 29˚C vs. JB197 37˚C (sheet labelled JB197). HB203 29˚C vs. HB203 37˚C was mapped to the JB197 genome (sheet labelled HB203_JBC), and HB203 29˚C vs. HB203 37˚C was mapped to the HB203 genome to include unique HB_C sRNA tags (sheet labelled HB203_HBC). Rows highlighted in orange represent significantly DE sRNAs (adjusted p value < 0.05 and a minimum fold change (FC) of three (Log2FC (1.585)).

Supplementary Material 5: Supplementary Table 3: Conservation of novel sRNAs. Summary of GLASSgo detected sRNA homologies found within the comprehensive Leptospiral genomes database. AccessionCt and taxonCt contain the count of unique NCBI accessions and NCBI taxonomic IDs, respectively, that contained a GLASSgo identified homolog of the query sRNA. The minimum, average, median, and maximum percent identity (PI) for the group was calculated across all GLASSgo identified homologs for the sRNA.

## Data Availability

All data is publicly available at NCBI (https://www.ncbi.nlm.nih.gov/geo/query/acc.cgi?acc=GSE168507) and Figshare (10.6084/m9.figshare.30066829).
