# Supplementary figures and images for: Identification and characterization of small RNAs expressed by Leptospira borgpetersenii serovar Hardjo and their conservation in the genus Leptospira
Source: BMC Microbiol. 2026 Apr 18;26:516. doi: 10.1186/s12866-026-05052-1 (PMC13224635; doi:10.1186/s12866-026-05052-1)

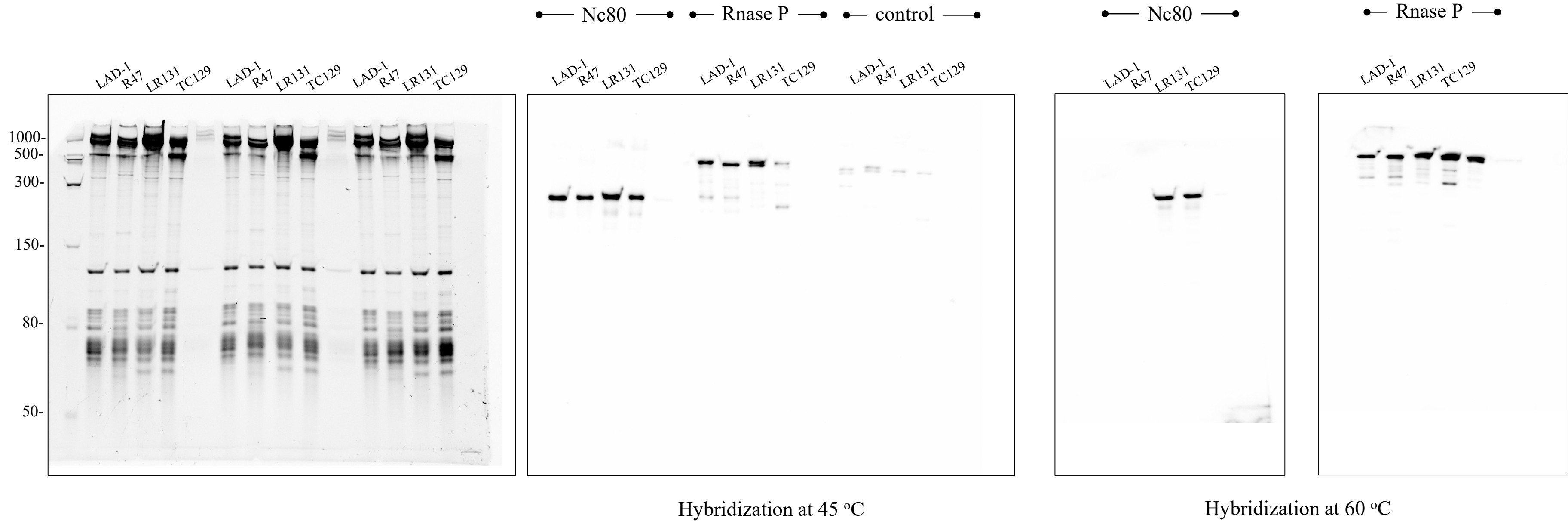

Supplement: Supplementary file 1 — Supplementary Material 1: Supplementary Fig 1: Original images of Northern Blots presented in Figure 2. [file 12866_2026_5052_MOESM1_ESM.pdf]
