# Supplementary material for: Identification and characterization of small RNAs expressed by Leptospira borgpetersenii serovar Hardjo and their conservation in the genus Leptospira
Source: BMC Microbiol. 2026 Apr 18;26:516. doi: 10.1186/s12866-026-05052-1 (PMC13224635; doi:10.1186/s12866-026-05052-1)

HB_C1_N1300

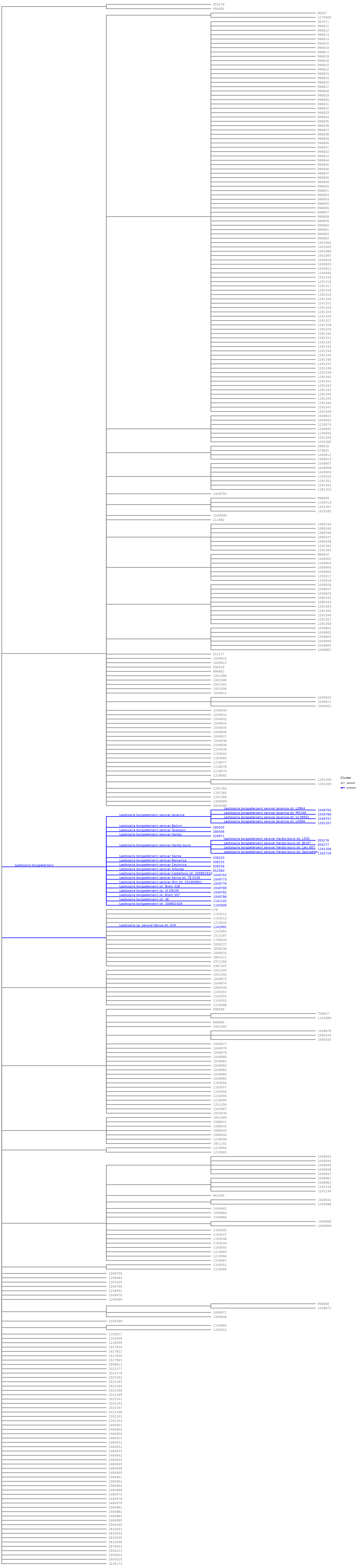

HB_C1_N1320

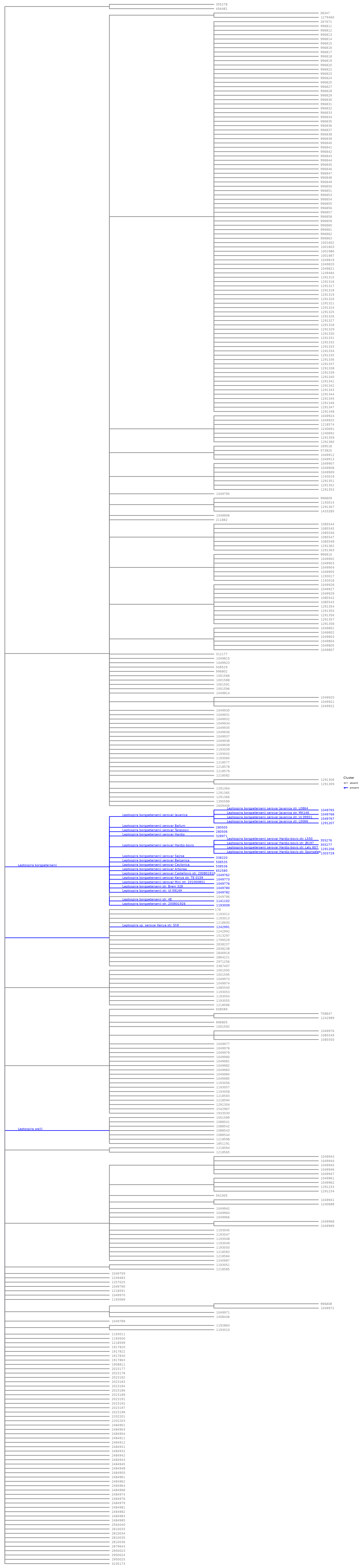
Leptospira borgpetersenii
Leptospira weilii
Cluster
absent
present

JB_C1_N135

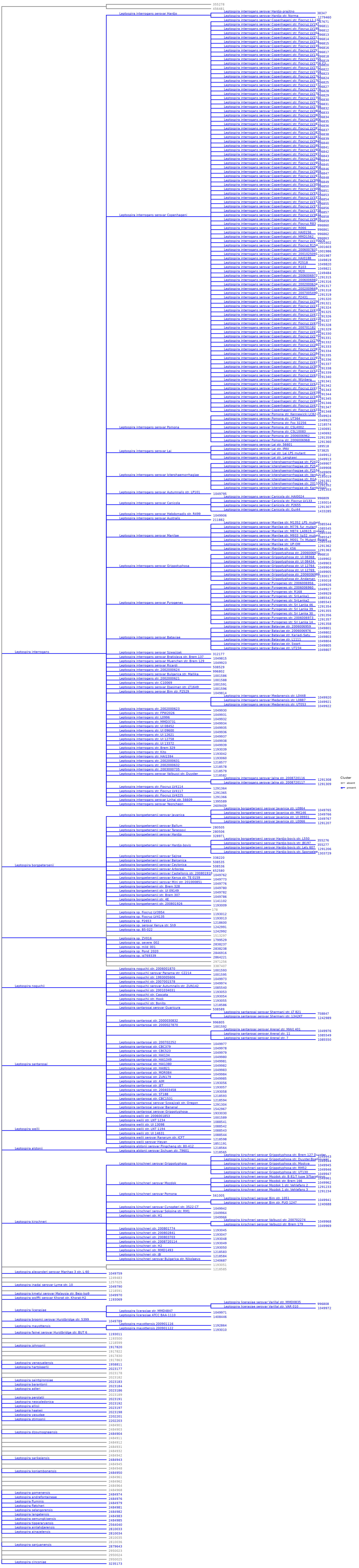

JB_C1_N495

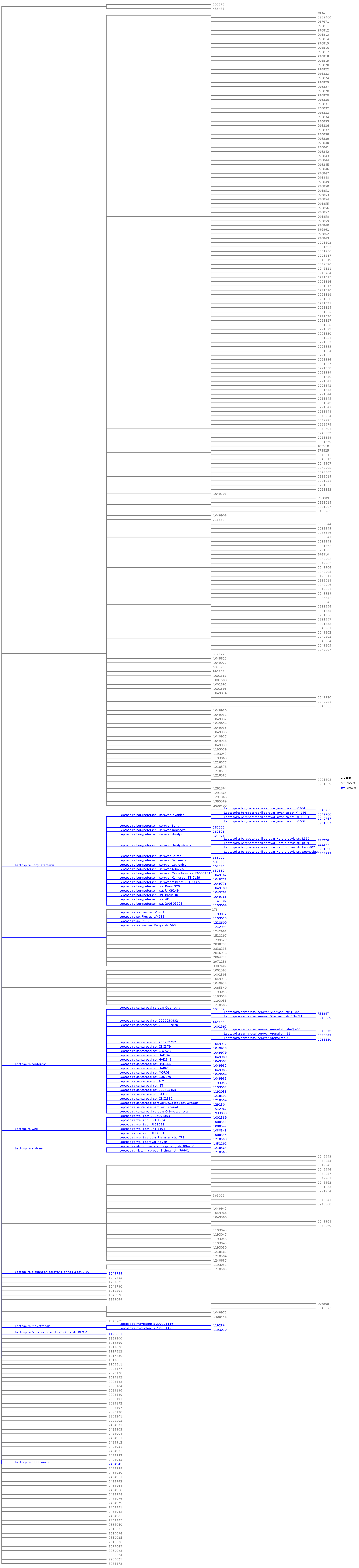

JB_C1_N530

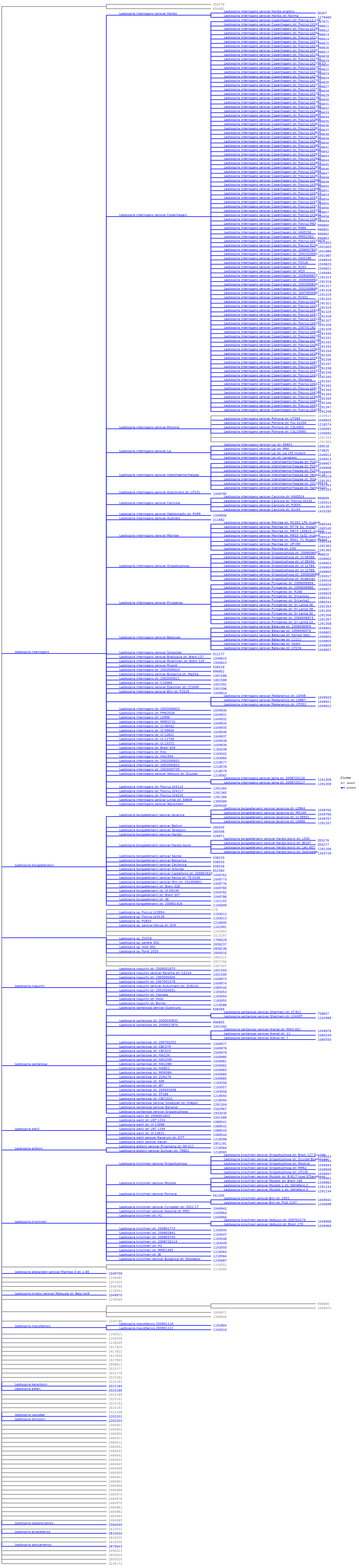

JB_C1_N595

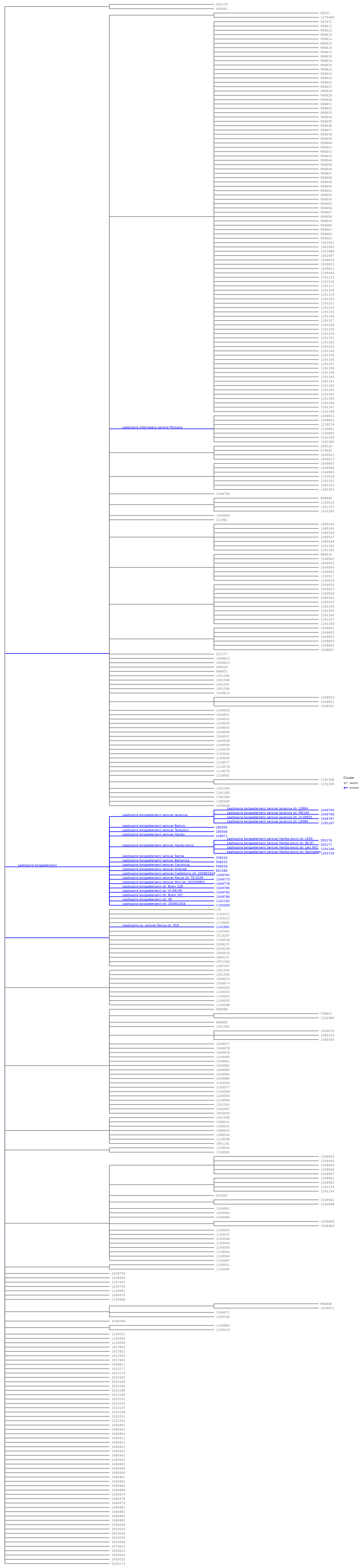

JB_C1_N610

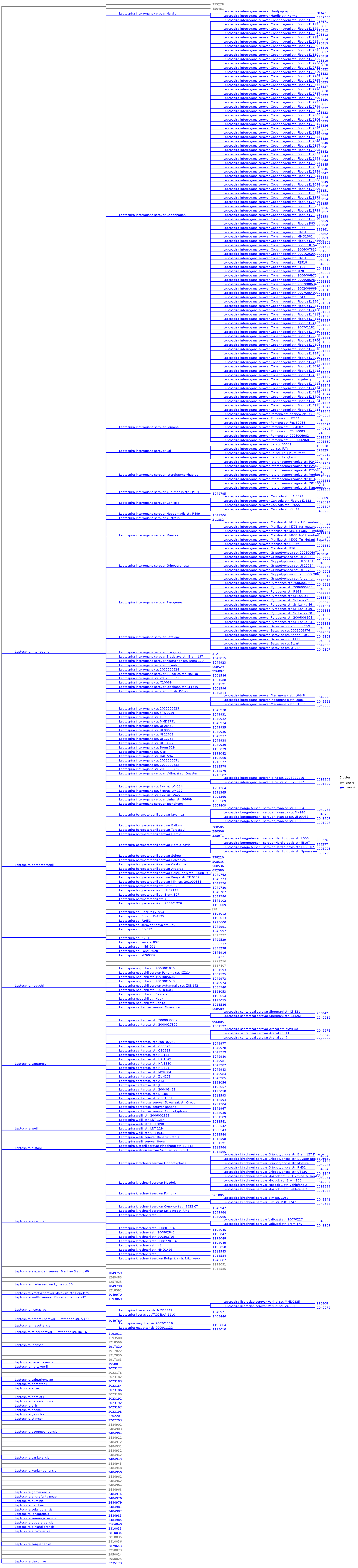

JB_C1_N645

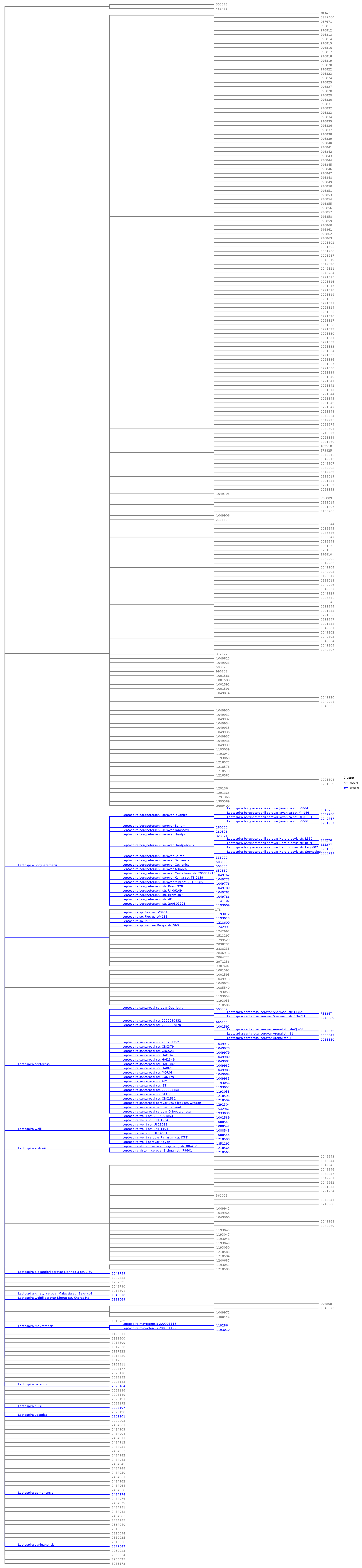

JB_C1_N725

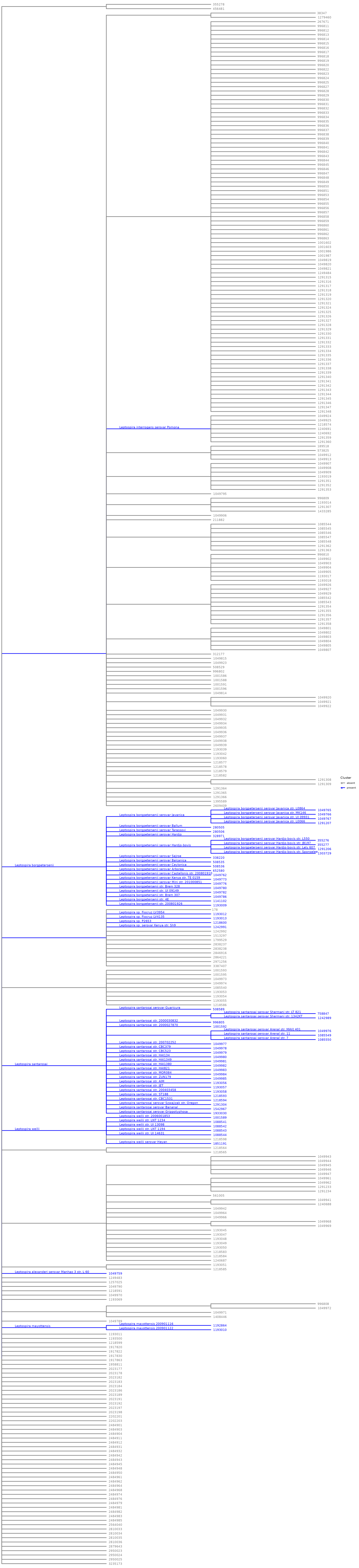

JB_C1_N790

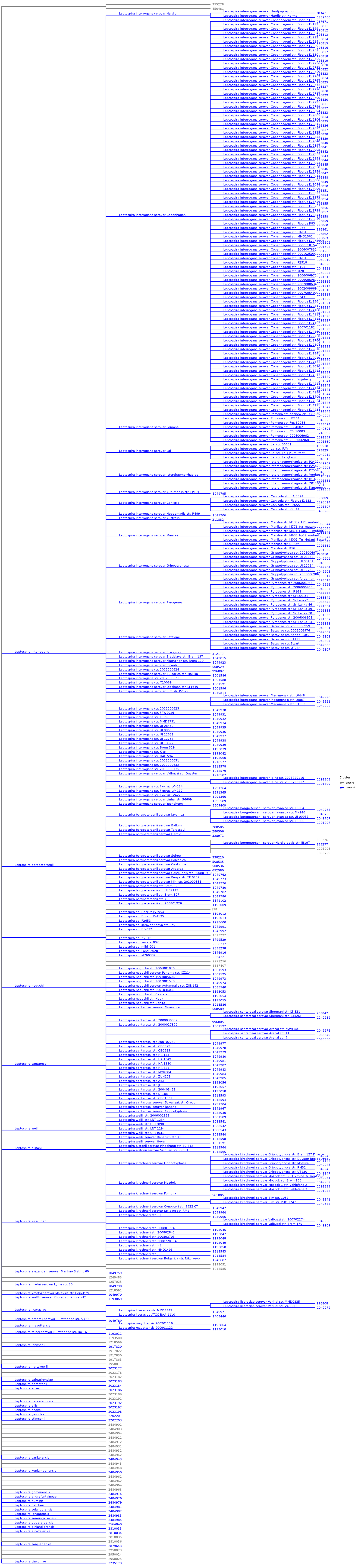

Supplement: Supplementary file 2 — Supplementary Material 2: Supplementary Fig. 2: NCBI taxonomic trees highlighting the presence of homologs for each sRNA listed in Table 2. The complete set of NCBI taxonomic trees highlighting the presence of homologs for all candidate sRNAs can be found at https://doi.org/10.6084/m9.figshare.30066829. [file 12866_2026_5052_MOESM2_ESM.pdf]
